# Exploring neural oscillations in numerical inductive reasoning: unveiling effects of top-down and bottom-up conflict

**DOI:** 10.3389/fpsyg.2023.1288325

**Published:** 2024-01-11

**Authors:** Shangqing Yuan, Jun Zhang, Tie Sun

**Affiliations:** ^1^School of Psychology, Research Center for Child Development, Beijing Key Laboratory of Learning and Cognition, Capital Normal University, Beijing, China; ^2^College of Home Economics, Hebei Normal University, Shijiazhuang, China; ^3^Joint Education Institute of Zhejiang Normal University and University of Kansas, Zhejiang Normal University, Jinhua, China; ^4^College of Education, Zhejiang Normal University, Jinhua, China

**Keywords:** numerical inductive reasoning, top-down conflict, bottom-up conflict, theta synchronization, alpha desynchronization

## Abstract

Previous research has delved into the brain’s response to top-down and bottom-up conflicts in numerical inductive reasoning. However, the specific neural oscillatory patterns associated with these conflict types in numerical inductive reasoning processing have remained elusive. In this study, we employed a number series completion task in which participants had to determine whether a given target number adhered to concealed rules. Three conditions were established: an identity condition (e.g., 13, 13, 13), a perceptual mismatch condition (representing bottom-up conflict, e.g., 13 13 十三), and a rule violation condition (representing top-down conflict, e.g., 13 13 14). Our EEG results revealed significant distinctions: rule violation induced more pronounced alpha desynchronization compared to both perceptual mismatch and identity conditions. Conversely, perceptual mismatch was associated with increased theta synchronization in contrast to rule violation and the identity condition. These findings suggest that alpha desynchronization may indicate the integration of rules during top-down conflict, while theta synchronization may function as a mechanism to inhibit bottom-up perceptual interference in numerical inductive reasoning.

## Introduction

Numerical inductive reasoning, as the core component of intelligence, could be affect by a myriad of factors (Oberauer et al., 2008). This form of reasoning is pivotal in understanding and manipulating numerical concepts, a skill fundamental in various cognitive tasks ranging from basic arithmetic to complex problem-solving. Recent studies have begun to unravel the neural underpinnings of this complex cognitive process, focusing particularly on the brain’s response to top-down (expectancy violation) and bottom-up (perceptual mismatch) conflicts (Liang et al., 2007; Qin et al., 2009; Li et al., 2012; Feng et al., 2014; Xiao et al., 2018, 2019).

Numerical inductive reasoning critically depends on the integration of numerical relationships among numbers for the extraction and application of rules. Research has shown that both numerical disparities and perceptual mismatches between adjacent numbers can significantly influence this process. For example, studies utilizing event-related potentials (ERP) have revealed that deviations in numerical value from preceding numbers elicit a more pronounced N200 component, as observed in research by Kong et al. (2000). Zhou et al. (2006) demonstrated that numbers exhibiting narrower value disparities between consecutive elements evoke larger N240 component amplitudes. This indicates that such disparities evoke an endogenous mismatch, conflicting with prior numerical representations and thereby demanding increased attention and working memory resources for the integration of these relationships. Moreover, perceptual mismatches have been noted to trigger N200, N400, and P300 components, highlighting the detection of variations in perceptual templates and the subsequent attentional requirements to inhibit processing of irrelevant conflicts during numerical tasks (Xiao et al., 2020). This underscores the significant impact of perceptual mismatches on the integration of numerical relationships.

Previous studies have demonstrated that expectation violations significantly affect the integration of numerical relationships, thereby impacting the process of numerical inductive reasoning. Research employing functional Magnetic Resonance Imaging (fMRI) and ERP techniques has shown that such violations activate key brain regions, including the dorsolateral prefrontal cortex (DLPFC), inferior parietal lobule (IPL), and frontal-polar cortex (FPC). The activation of these areas reflects changes in hypotheses, computational demands, and the incorporation of incongruent rules (Feng et al., 2014). Moreover, expectation violations have been found to elicit specific neural responses, such as the N200, P300, and late positive component (LPC), as identified by Xiao et al. (2018). These components primarily signal the detection of conflict between expected and actual numbers, a sense of uncertainty, and the necessity for working memory updating to integrate these numerical relationships.

A recent investigation aimed to distinguish between top-down (expectation violation) and bottom-up (perceptual mismatch) conflicts in the context of numerical inductive reasoning (Xiao et al., 2019). In this study, top-down conflict involves a violation of rule expectations, compelling participants to reassess established rules and consciously apply top-down cognitive control to integrate numerical relationships. Conversely, bottom-up conflict is characterized by perceptual mismatches, where participants must filter out irrelevant perceptual information, thus requiring heightened sensitivity to distracting stimuli. While both types of conflict involve some form of change, rule violations are specifically associated with top-down conflict due to the need for rule integration. In contrast, perceptual mismatches embody bottom-up conflict, focusing on the management of interference from non-essential perceptual changes. The findings of this study revealed that perceptual mismatches trigger neural responses similar to those of rule violations, including P200, N200, P300, and LPC components, which are indicative of attention reallocation, deviations in perceptual templates, feelings of uncertainty, and the need for working memory updating, respectively. Notably, it was the rule violation condition that uniquely elicited the N400 and late negative component (LNC). This suggests that alterations in number values, as a form of rule violation, engage top-down conflict by challenging established rule expectations.

Previous research has primarily concentrated on exploring the temporal dynamics of top-down and bottom-up conflicts in numerical inductive reasoning. However, reliance on ERP analysis might lead to an incomplete understanding of certain task-related cognitive processes. This is because traditional ERP analysis might miss critical information within time-locked and non-phase-locked activities, which are essential for a comprehensive grasp of cognitive processing. As a result, ERP studies, such as those conducted by Xiao et al. (2019), may not have fully captured all the cognitive processes associated with top-down and bottom-up conflicts in this context. The current study aims to bridge this gap by delving deeper into the cognitive processes tied to these conflicts, employing neural oscillation measurements and utilizing time-frequency analysis techniques to detect changes in power across various frequency bands.

The objective of the present study is to delineate the distinct neural oscillatory patterns associated with top-down and bottom-up conflicts during numerical inductive reasoning, particularly focusing on the theta and alpha frequency bands. Theta band synchronization, ranging from 4 to 8 Hz, is acknowledged for its vital role in various cognitive functions, including fostering focused attention (Ishii et al., 1999), responding to rule violations (Tzur and Berger, 2007), and facilitating cognitive control from diverse conflicts, even those involving task-irrelevant information (Nigbur et al., 2011). In contrast, the alpha band, spanning 8 to 13 Hz, is known to exhibit desynchronization during cognitive tasks such as mental arithmetic (De Smedt et al., 2009), reasoning (Neubauer and Fink, 2003), and memory-related processes (Krause et al., 2000). Additionally, alpha band desynchronization has been linked to the redirection of attention towards task-specific information processing, as highlighted in the research by Klimesch (1997).

In the current study, our aim is to uncover the additional cognitive processing associated with top-down and bottom-up conflicts during numerical inductive reasoning. We seek to extend the findings obtained through ERP-based results by examining neural oscillation. To achieve this goal, we reanalyzed the data from Xiao et al. (2019). This study employed the number series completion task, generating three distinct experimental conditions: (a) an identity condition with no rule or perceptual changes (e.g., “1, 1, 1”); (b) a rule-violation condition involving a change in rules without perceptual alterations (e.g., “1, 1, 2”); and (c) a perceptual mismatch condition featuring a perceptual change while maintaining the rule (e.g., “1, 1, 一”). We hypothesized that, compared to the identity condition, the rule violation condition (representing top-down conflict) would result in increased theta synchronization and alpha desynchronization. As the third number in the rule violation condition semantically diverges from the standard sequence, this more pronounced conflict may require additional cognitive resources to detect the violation and integrate higher-order relationships (Neubauer and Fink, 2003; Tzur and Berger, 2007), necessitating a reallocation of attention, updating of working memory, detection of expectancy violations, and enhanced cognitive control. Additionally, we anticipated that the perceptual mismatch condition (embodying bottom-up conflict) would induce greater theta synchronization compared to the identity condition, as the perceptual change in the third number represents a task-irrelevant conflict. This aligns with the known correlation between theta synchronization and the inhibition of distractions (Nigbur et al., 2011).

## Methods

### Participants

Twenty-five right-handed students (14 males, aged 23.76 ± 2.32 years) from Shanxi Normal University participated in the present study. The EEG data of one participant was excluded from our analysis due to an unacceptable level of signal noise and artifacts. None of the participants had a history of neurological or psychiatric disorders, and all had normal or corrected-to-normal vision. The study received ethical approval from the local ethics committee, and informed consent was obtained from each participant.

### Experimental design

**Figure 1:**
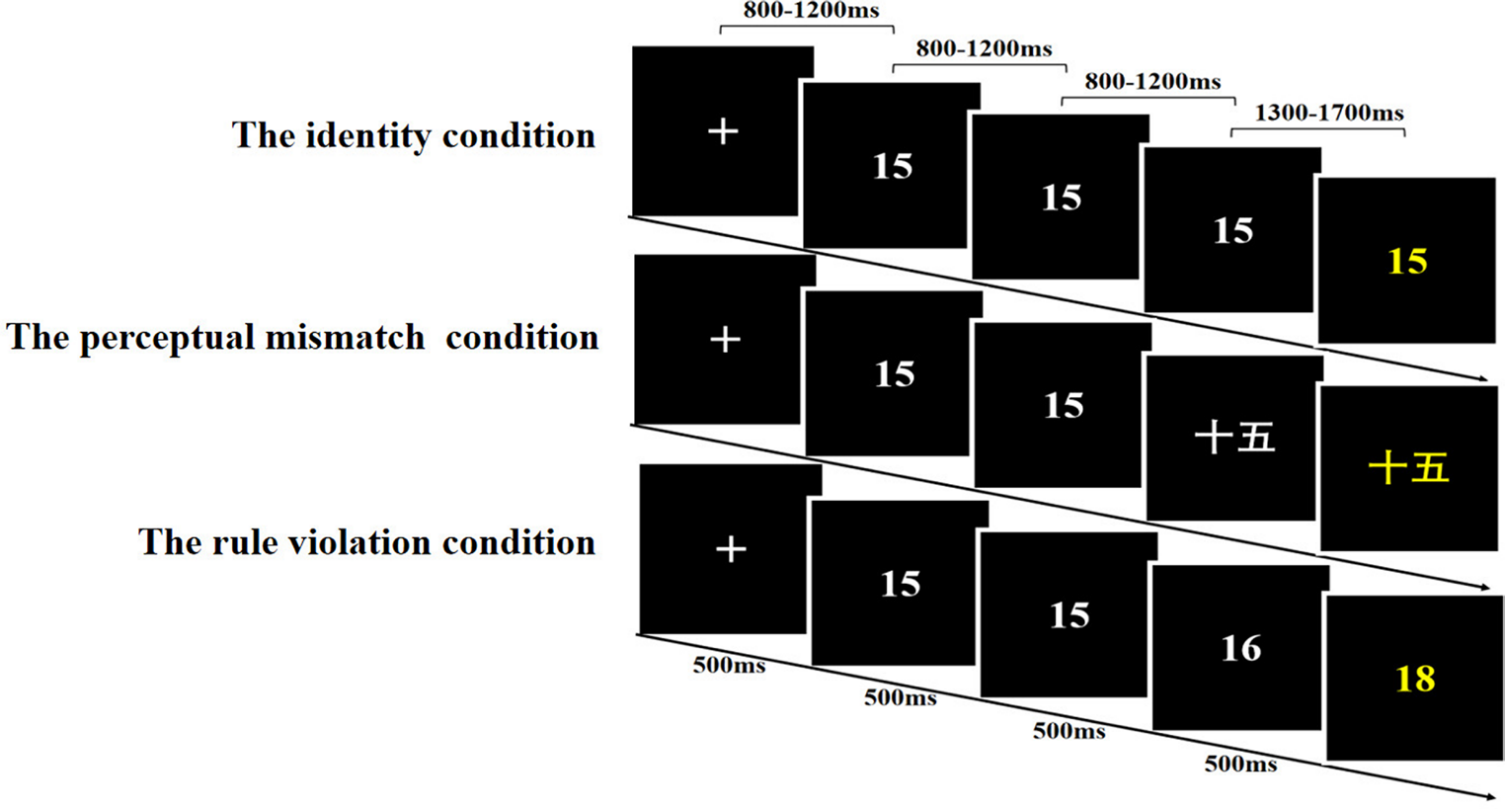
Experimental procedures for the number series completion task.

In the present study, three conditions were created, each consisting of 80 trials presented randomly. As depicted in Figure 1, four numbers in each trial were sequentially presented, and participants were required to determine whether the fourth number was congruent with the hidden rules. In each trial, a set of four numbers was displayed against a black background, rendered in a 38-point Courier New font. The initial three numbers were displayed in white, while the fourth number, serving as the probe, was distinctly highlighted in yellow. In the identity condition, without any perceptual or rule changes, the four numbers were the same (e.g., “15, 15, 15,” with the rule being +0). In the perceptual mismatch condition, the only perceptual change in the third number could evoke bottom-up conflict in inductive reasoning (e.g., “15, 15, 十五,” with the rule being +0). In the rule violation condition, the only rule change occurred in the third number, which could elicit top-down conflict in inductive reasoning (e.g., “15, 15, 16,” with the rule being +0, +1). All values within the series remained below 20, and the operands in the rule violation condition changed gradually, varying from ±1 to 8. The pattern of numerical rules and the range of values in the experiment were explained to the participants during the practice stage, where they were allowed to practice and become familiar with the rules.

The formal experimental procedure, as depicted in Figure 1, began with the screen initially displaying a crosshair at the center for a duration of 500 milliseconds (ms). This was followed by the sequential presentation of three numbers, each shown for a period of 500 ms. These number presentations were separated by blank intervals ranging from 800 to 1,200 ms. Subsequently, a blank period lasting between 1,300 and 1700 ms ensued, after which the target number was presented. The probe number, once presented, remained visible for a duration of 2000 ms. During this period, participants were tasked with determining whether the target number adhered to the established rule of the number sequence. Their response was elicited using the F (or J) key within a 2000 ms timeframe. Notably, the experimental design maintained a 1:1 ratio between correct and incorrect answers, and the assignment of response keys was counterbalanced across all participants. Throughout the experiment, the participants’ eyes were approximately 60 cm away from the screen.

### Electrophysiological recording and analysis

The recording of EEG data was conducted using an electrode cap and data recording software manufactured by Brain Products GmbH, Germany. EEG recordings were performed using a 64-channel electrode cap following the international standard 10–20 system. During data recording, FCz was employed as the reference channel, while Afz was designated as the ground. Two electrodes were positioned above and below the right eye (EOG) to capture vertical eye movement. The EEG data were collected online at a sampling rate of 500 Hz, with a bandpass filter range of 0.05-100 Hz. The experiment commenced once the electrode impedance across all points fell below 5 KΩ.

For offline analysis, the EEGLAB analysis package was employed for data preprocessing. Bilateral mastoid electrodes (TP9, TP10) were selected as reference electrodes. Independent Component Analysis (ICA) was used to remove noise sources, including ocular artifacts and other artifacts. A high-pass filter was set at 0.1 Hz, and a low-pass filter was set at 40 Hz. Data segments were extracted from 800 ms prior to the baseline to 2000 ms after stimulus presentation, with each segment lasting a total of 2,800 ms.

The preprocessed data underwent time-frequency analysis using the Morlet wavelet transform method. The frequency range analyzed spanned from 3.9 to 40 Hz, and 6 cycle numbers were linearly divided into 50 frequency points, resulting in the generation of a 2D matrix data of 40 (frequency points) × 1,400 (time points) for each trial. The time window for baseline correction extended from 200 ms to 50 ms before stimulus onset, and the energy magnitude was transformed into decibel (dB) scale for normalization: dB = 10 × log10(μV^2). Task-associated power at a specific electrode was quantified by subtracting dB values obtained during the baseline interval from those acquired during the presentation of the third number. In this context, positive values signify that oscillations within the activation interval surpass baseline levels, indicative of event-related synchronization (ERS), while negative values indicate that oscillations within the activation interval fall below the baseline, characterizing event-related desynchronization (ERD).

Based on visual inspection, theta band (4-8 Hz) oscillations were observed across all electrodes approximately 0-1000 ms after the presentation of the third number in all three conditions. Alpha band (8-13 Hz) oscillations emerged around 0-1000 ms after the third number presentation in the identity and perceptual mismatch conditions, and within 0-1400 ms in the rule violation condition. Following Xiao et al. (2018), a total of nine electrodes were selected, encompassing the frontal (F3, FZ, F4), central (C3, CZ, C4), and parietal (P3, PZ, P4) regions. A two-factor repeated-mearsures ANOVA (9 Electrode sites × 3 conditons) was conducted to elucidate variations among these three conditions during two distinct time windows: 0-1000 ms and 1,000-1400 ms. The *p-*values were corrected using the Greenhouse–Geisser method, and the *post hoc* tests were corrected using the Bonferroni method.

## Results

### Behavioral results

The mean accuracy values for responses towards target numbers were calculated as follows: 0.98 ± 0.01 for the identity condition (mean ± standard error), 0.96 ± 0.01 for the perceptual mismatch condition, and 0.95 ± 0.11 for the rule violation condition. A repeated measures ANOVA revealed a significant main effect of condition [*F* (2, 48) = 5.14, *p* = 0.028, *η_p_*^2^ = 0.18]. *Post hoc* tests demonstrated that accuracy was significantly higher in the identity condition compared to the other two conditions (*ps* ≤ 0.018), while no significant difference was found between the perceptual mismatch and rule violation conditions (*p* = 0.136).

Focusing on data exclusively from correct trials, response times (RTs) across the three conditions were collected for target number stimuli. The mean RTs were 750 ± 44 ms for the identity condition, 790 ± 52 ms for the perceptual mismatch condition, and 1,487 ± 181 ms for the rule violation condition. The main effect of condition was found to be statistically significant [*F* (2, 48) = 22.13, *p* < 0.001, *η_p_*^2^ = 0.48]. Subsequent *post hoc* analyses indicated that the rule violation condition exhibited significantly longer RTs compared to the other two conditions (*ps* < 0.001), while no significant difference was observed between the identity condition and the perceptual mismatch condition (*p* = 0.086).

### EEG results

#### Theta (4-8 Hz) ERS

**Figure 2:**
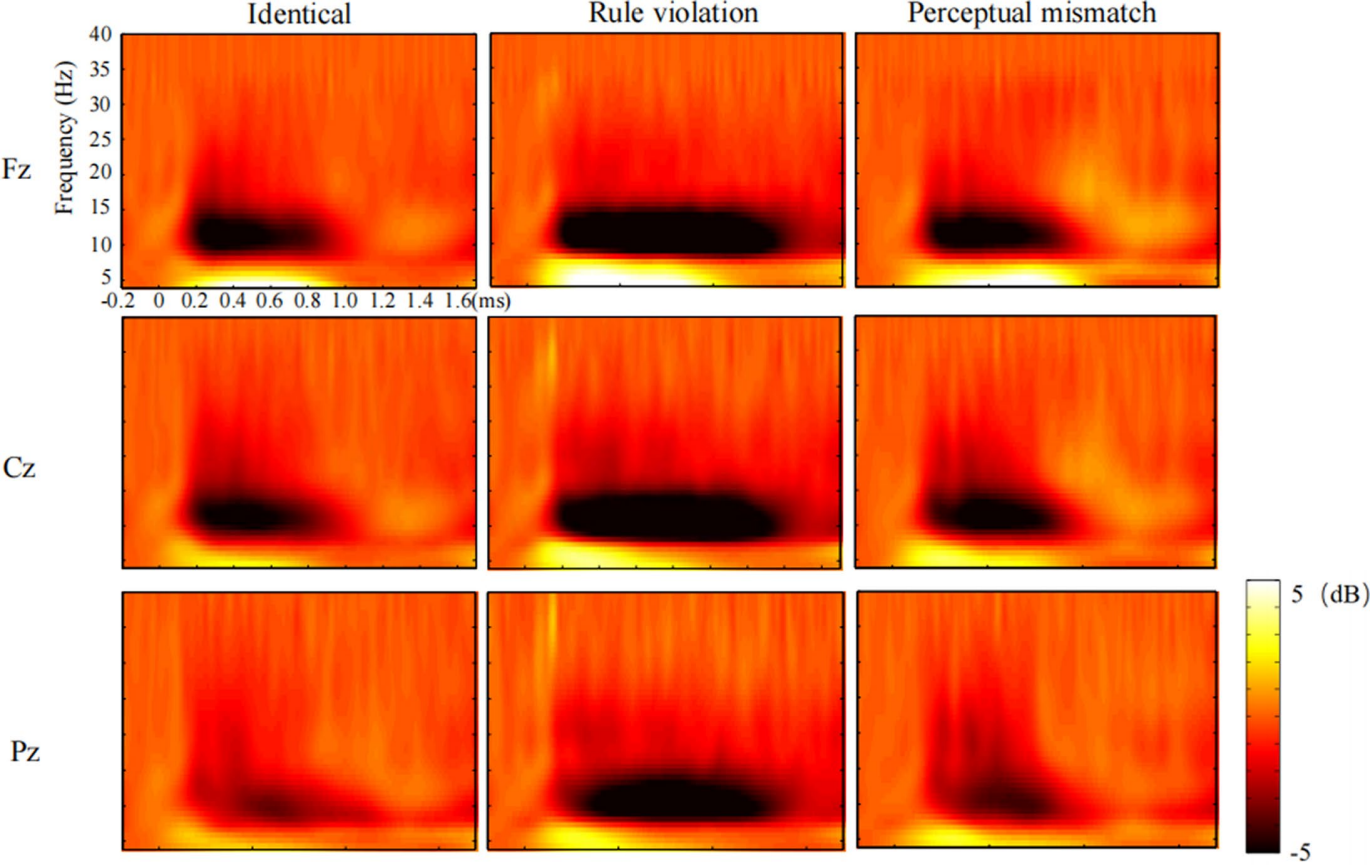
Time-frequency power maps for identity, rule violation and perceptual mismatch conditions in three channels (Fz, Cz, Pz).

For the total theta power of the third numbers in 0-1000 ms time window, as shown in the Figure 2, the main effect of condition was significant [*F* (2,46) = 6.71, *p* = 0.007, *η_p_^2^* = 0.23]. The *post hoc* test showed that the perceptual mismatch condition evoked larger theta band power than both the identity condition (*p* = 0.001) and the rule violation condition (*p* = 0.022), with no significant difference observed between the identity and rule violation condition (*p* = 1.000). Additionally, the main effect of site on theta was significant [*F* (8,184) = 78.14, *p* < 0.001, *η_p_^2^* = 0.77], and the interaction effect of Condition × Site was not significant [*F*(16, 368) = 1.88, *p* = 0.137, *η_p_^2^* = 0.08].

For the total theta power of the third numbers in 1000-1400 ms time window, as shown in the Figure 2, the main effect of condition was not significant [*F* (2,46) = 0.20, *p* = 0.677, *η_p_^2^* = 0.01]. Besides, the main effect of site on theta [*F* (8,184) = 6.14, *p* = 0.003, *η_p_^2^* = 0.211] and the interaction effect of Condition × Site [*F*(16, 368) = 3.17, *p* = 0.019, *η_p_^2^* = 0.12] were both significant.

#### Alpha (8-13 Hz) ERD

For the total alpha power of the third numbers in 0-1000 ms time window, as shown in the Figure 2, the main effect of condition was significant [*F* (2.46) = 12.53, *p* < 0.001, *η_p_^2^* = 0.35]. The *post hoc* test indicated that the rule violation condition evoked larger alpha band power than both the identity (*p* = 0.002) and perceptual mismatch (*p* = 0.004) conditions, with no significant difference found between the identity and perceptual mismatch conditions (*p* = 1.000). Additionally, the main effect of site on alpha [*F* (8,184) = 2.23, *p* = 0.119, *η_p_^2^* = 0.09] and the interaction effect of Condition × Site [*F*(16, 368) = 1.06, *p* = 0.386, *η_p_^2^* = 0.04] were not significant.

For the total alpha power of the third numbers in 1000-1400 ms time window, as shown in the Figure 2, the main effect of condition was significant [*F* (2.46) = 25.43, *p* < 0.001, *η_p_^2^* = 0.53]. The *post hoc* test revealed that the rule violation condition evoked larger alpha band power than both the identity and perceptual mismatch conditions (*ps* < 0.001), with no significant difference noted between the identity and perceptual mismatch conditions (*p* = 0.190). Besides, the main effect of site on alpha was not significant [*F* (8,184) = 0.71, *p* = 0.543, *η_p_^2^* = 0.03], and the interaction effect of Condition × Site was significant [*F*(16, 368) = 5.51, *p* < 0.001, *η_p_^2^* = 0.19].

## Discussion

The present study aimed to dissociate the neural oscillatory characteristics associated with top-down and bottom-up conflicts inherent in numerical inductive reasoning. These conflicts were manipulated through rule violations and perceptual mismatches. The behavioral outcomes demonstrated that participants performed less accurately in the rule violation and perceptual mismatch conditions, accompanied by longer response times in rule violation condition than the identity condition. These behavioral findings substantiate the impact of these conflict types on numerical inductive reasoning. Furthermore, EEG results revealed that within the 0-1000 ms time window, rule violation condition induced greater alpha desynchronization than perceptual mismatch and identity conditions. The perceptual mismatch condition was associated with increased theta synchronization compared to rule violation and the identity condition. Additionally, in the 1,000-1400 ms time window, there was only increased alpha desynchronization in the rule violation condition. These findings indicate that in numerical inductive reasoning, theta synchronization appears to be particularly responsive to bottom-up conflict, whereas alpha desynchronization exhibits a particular sensitivity to top-down conflict.

The present study revealed that the perceptual mismatch condition elicited larger theta synchronization in comparison to both the identity and rule violation conditions. This finding underscores the association between theta synchronization and the processing of task-irrelevant perceptual information in numerical inductive reasoning. This result aligns with previous studies, in which theta synchronization has consistently been linked to situations involving interference. For instance, in tasks such as the go/nogo task, increased theta synchronization was observed in the nogo condition, which necessitated response inhibition (Yamanaka and Yamamoto, 2010). Similarly, in the flanker task, researchers noted increased theta synchronization in incongruent conditions, indicating that theta synchronization is sensitive to distracting information (Nigbur et al., 2011). In the present study, participants were tasked with determining whether the target number was congruent with the hidden rules. To achieve this objective, participants needed to focus on numerical values, rendering perceptual changes as task-irrelevant information. The evocation of theta synchronization in response to this bottom-up conflict aligns with the role of theta synchronization in processing distractions.

However, the present study did not uncover increased theta synchronization induced by the rule violation condition. A previous study adopted a simple mathematical equation task and found that incorrect solutions elicited larger theta synchronization than correct solutions, with greater deviation between incorrect and correct answers corresponding to higher theta synchronization (Tzur and Berger, 2007). Consequently, theta synchronization was predominantly linked to the detection of incongruent information. In the present study, the numerical alterations in the third number of the sequence entailed not only the processing of incongruent information but also demanded a higher-level integration of rules. This resulted in the absence of increased theta synchronization in the rule violation condition.

Furthermore, the present study revealed that the rule violation condition exhibited a greater degree of alpha desynchronization compared to the other two conditions. This result aligns with findings from previous studies, where enhanced alpha desynchronization occurs during cognitively demanding tasks as opposed to the rest state and simple tasks. For instance, in a visual letter n-back task, increased alpha desynchronization was observed during the 2-back condition, signifying that the augmented memory load led to a more pronounced alpha desynchronization (Krause et al., 2000). Similarly, previous studies demonstrated that in the Stankov’s Triplet Numbers test, heightened task complexity was associated with increased alpha desynchronization (Neubauer and Fink, 2003). In the present study, the numerical alteration in the third number, which disrupted the rule established through the initial two numbers, prompted a top-down conflict that necessitated participants to integrate numerical relationships and revise their rule structure. In contrast to the identity and perceptual mismatch conditions, this process in rule violation condition exhibited greater complexity and required more cognitive resources, resulting in a more substantial alpha desynchronization.

The present findings also suggest a flexible attention regulation mechanism during numerical inductive reasoning. Both the rule violation and perceptual mismatch conditions involved changes in perceptual characteristics, yet they elicited distinct responses in EEG frequency bands. Specifically, in the rule violation condition, both the shape and value of the numbers changed. However, since this study only required participants to judge numerical rules, their attentional resources were primarily focused on processing conflicts with expected values, thereby inducing alpha oscillations linked to task-related processing. In contrast, under the perceptual mismatch condition, perceptual changes constituted a bottom-up conflict, leading participants to redirect attentional resources toward processing task-irrelevant information, thus inducing theta oscillations.

It is worth mentioning that the present study successfully dissociated distinct brain responses arising from top-down and bottom-up conflicts based on neural oscillatory features. Previous studies have investigated the brain response to top-down and bottom-up conflict underlying inductive reasoning, mainly focusing on the ERP components. They found that the top-down conflict specifically triggered the N400 and LNC, reflecting the detection of expectancy violations (Xiao et al., 2019). However, no distinct neural component was found to be specifically evoked by bottom-up conflict; the P200, N200, P300, and LPC were elicited by both bottom-up and top-down conflicts. The present study analyzed the neural oscillatory characteristics and revealed that the alpha desynchronization specific to rule violation reflects the integration of rule relationships as a top-down conflict, and the theta synchronization specific to perceptual mismatch reflects the inhibition of perceptual interference associated with bottom-up conflict.

Present study elucidates the neural mechanisms involved in resolving cognitive conflicts during numerical inductive reasoning, highlighting a flexible attentional regulation mechanism. The differential roles of theta and alpha oscillations in processing top-down and bottom-up conflicts are clarified, shedding light on how the brain allocates attention in various conflict scenarios. These findings carry important implications for education and cognitive training, laying the groundwork for future research into complex reasoning and decision-making processes.

### Limitations and future directions

The present study investigated the neural oscillatory patterns associated with top-down and bottom-up conflicts in numerical inductive reasoning. However, there are several limitations that warrant consideration in future studies. Firstly, the study’s sample size may limit the generalizability of the findings. Future research could benefit from incorporating larger and more diverse participant groups to ensure broader applicability. Additionally, the relatively simplified numerical reasoning task used in this study may not fully capture the complexities of real-world problem-solving scenarios. This highlights the importance of investigating more intricate reasoning processes. Furthermore, this study examined top-down and bottom-up conflicts in isolation. Real-world reasoning often involves the interplay of multiple conflict types. Future research could explore how these conflicts interact and influence neural oscillations, providing a more comprehensive understanding of the cognitive processes involved.

## Conclusion

The present study effectively disentangled the distinct brain responses arising from top-down and bottom-up conflicts in numerical inductive reasoning by analyzing neural oscillatory features. Through the manipulation of rule violations and perceptual mismatches, we observed behavioral changes reflected in decreased accuracy and prolonged response times, confirming the impact of these conflict types on numerical inductive reasoning. EEG results highlighted specific oscillatory patterns associated with different conflict types within specific time windows. Notably, theta synchronization exhibited sensitivity to bottom-up conflict, whereas alpha desynchronization was more responsive to top-down conflict. These findings contribute to a deeper understanding of the neural mechanisms underlying cognitive conflict resolution in numerical inductive reasoning and shed light on the role of neural oscillations in processing different types of conflicts.

## Data availability statement

The raw data supporting the conclusions of this article will be made available by the authors, without undue reservation.

## Ethics statement

The study was approved by the Ethical Committee of the Department of Education Science, Shanxi Normal University and all participants signed an informed consent document approved by the University’s Institutional Review Board.

## Author contributions

SY: Conceptualization, Writing – original draft, Writing – review & editing. JZ: Writing – review & editing. TS: Conceptualization, Data curation, Formal analysis, Writing – review & editing.
